# Distinct human small intestinal microbiome communities underlie visceral hypersensitivity in a humanized mouse model

**DOI:** 10.1172/JCI190638

**Published:** 2025-10-23

**Authors:** Isin Y. Comba, Tijs Louwies, Ruben A.T. Mars, Yang Xiao, Prabhjot Kaur Sekhon, Brian S. Edwards, Adam Willits, Robin R. Shields-Cutler, Shreya Bellampalli, Arnaldo Mercado-Perez, Dennis R. Tienter, Lisa M. Till, David R. Linden, Gianrico Farrugia, Arthur Beyder, Kristen M. Smith-Edwards, Purna C. Kashyap

**Affiliations:** 1 Mayo Clinic, Rochester, Minnesota, USA.; 2 Macalester College, St. Paul, Minnesota, USA.

**Keywords:** Gastroenterology, Microbiology, Bacterial infections, Mouse models, Pain

## Abstract

Specific small-intestinal bacteria, particularly *Enterobacteriaceae*, can trigger abdominal pain by activating gut sensory and nerve cells, revealing how microbiota contribute to gastrointestinal symptoms.

**To the Editor:** While gastrointestinal (GI) symptoms may stem from small intestinal bacterial overgrowth (SIBO), our recent work has shown a significant association between GI symptoms, such as bloating and abdominal pain, and small intestine (SI) microbial composition, rather than SIBO per se (1). Specific models to study the biological effects of SI microbes are lacking; thus, we sought to establish such a model. The current approach of colonizing germ-free (GF) mice with human fecal samples may not recapitulate the human small intestinal microbiome. Therefore, we colonized GF mice with human SI aspirates, fecal samples, or SI aspirates followed by fecal samples from the same individual (Figure 1A). SI microbiota β-diversity (Figure 1B) and α-diversity (Figure 1C) of GF mice colonized with human SI aspirates most closely resembled the diversity of human SI microbiota, compared with GF mice colonized with human feces or SI aspirate followed by feces. The engraftment percentages ranged from 46.4% to 70.0%, and the most abundant genus from the human SI donor sample was detected in every corresponding mouse SI (Supplemental Table 1; supplemental material available online with this article; https://doi.org/10.1172/JCI190638DS1). These findings establish a murine model of humanized SI microbiota and underscore the importance of employing site-specific input samples to investigate the impact of microbiota.

To determine whether human SI microbes specifically contribute to abdominal pain, we colonized GF mice with SI aspirates either from 3 healthy individuals (HC mice) or 3 patients with abdominal pain as the predominant symptom (AP mice) (donor characteristics are outlined in Supplemental Table 2) and measured visceromotor responses (VMRs) to colorectal distention (CRD), whole-gut transit, and intestinal permeability (Figure 1D). No differences were observed in whole-gut transit time or intestinal permeability (Supplemental Figure 1, A and B). AP mice had significantly higher VMRs to CRD compared with those of HC mice (Figure 1E, Supplemental Figure 2A, and Supplemental Table 3), indicating visceral hypersensitivity. These findings highlight that SI microbiome transplantation induces a donor-specific phenotype in recipient mice, reflecting the clinical presentation of patients with abdominal pain.

Enterochromaffin (EC) cells, which make up the largest population of enteroendocrine cells, are polymodal stimulus detectors known to transduce noxious stimuli from the colonic lumen to nearby nerve endings via the release of serotonin (5-HT) to induce visceral hypersensitivity (2). To test if EC cells similarly act as signal transducers in the SI, we measured whether SI aspirates from AP and HC mice triggered Ca^2+^ responses in QGP-1 cells (EC cell model) and SI organoids made from NeuroD1-cre;GCaMP5-tdTomato mice (3, 4). We found that SI aspirates from AP mice evoked significantly higher Ca^2+^ responses in QGP-1 cells and SI organoids when compared with HC mice (Figure 1, F and G; Supplemental Figure 2, B and C; and Supplemental Table 3). As some microbial products can diffuse or be transported across the epithelium, we also tested the ability of SI aspirates to directly activate neurons from the thoracic (T8–T11) dorsal root ganglia (DRG) (5). Interestingly, SI aspirates from AP mice activated significantly more DRG neurons and triggered higher Ca^2+^ responses in these neurons when compared with HC mice (Figure 1, H and I; Supplemental Figure 2A; and Supplemental Table 3). Altogether, these findings suggest that SI contents from AP mice may engage pain pathways indirectly by activating EC cells or directly by activating DRG neurons (2).

To identify specific microbes associated with visceral hypersensitivity in AP mice, we analyzed the SI microbiota of a patient with abdominal pain, 2 healthy individuals acting as controls, and their corresponding mouse SI aspirates. *Blautia*_ASV27 and *Enterobacteriaceae*_ASV5 were significantly enriched in AP mice and were detected solely in the patient with abdominal pain. Interestingly, *Enterobacteriaceae*_ASV5 exhibited a positive correlation with the VMR at 30 mmHg (adjusted R^2^ = 0.49, *P* = 0.01, linear regression). To determine whether the *Enterobacteriaceae* family is more broadly associated with abdominal pain or bloating, we reanalyzed previously published small intestinal microbiome sequencing data (1) from 98 study participants and found that the relative abundance of *Enterobacteriaceae* was higher in patients with abdominal pain or bloating compared with healthy individuals acting as controls (Figure 1J). To further determine whether specific strains within the *Enterobacteriaceae* family might be driving visceral pain sensation, we cultured one AP SI sample on *Enterobacteriaceae*-selective media and obtained multiple colonies, all of which were found to be the same strain of *Enterobacter hormaechei* through MALDI and Genome Taxonomy Database Toolkit analysis of the assembled genome (Supplemental Table 4) (6).

We repeated our earlier experiments with culture supernatants from the isolated *E*. *hormaechei* strain. As controls, we used the Gram-positive *Streptococcus oralis* ATCC9811, which closely matched the most abundant amplicon sequence variant (ASV) in the HC mice, and a clinical strain of the Gram-negative Proteobacterium *Aggregatibacter aphrophilus* (previously called *Haemophilus aphrophilus*), which broadly shares the lipopolysaccharide architecture with *Enterobacter* but is not a member of the *Enterobacteriaceae*. *E*. *hormaechei* supernatants elicited significantly higher Ca^2+^ responses in QGP-1 cells and activated significantly more and elicited higher Ca^2+^ responses in DRG neurons when compared with supernatants from *S*. *oralis* and *A*. *aphrophilus* (Figure 1, K–M, and Supplemental Table 5). Additionally, GF mice colonized with *E*. *hormaechei* exhibited significantly higher VMRs to CRD compared with GF mice and GF mice colonized with *S*. *oralis* or *A*. *aphrophilus*, phenocopying the responses from entire SI aspirates (Figure 1N).

Our findings underscore the previously unrecognized significance of specific human SI communities in inducing GI symptoms. Although further validation in larger studies is warranted, our results suggest that specific members of *Enterobacteriaceae* within SI microbiome are likely important players in generation of GI symptoms. Additionally, this work advances our understanding of how microbial products interact with the SI sensory function and opens new avenues of research into the SI sensory apparatus and its communication with the brain.

For detailed methods, information regarding sex as a biological variable, statistics, study approval, data availability, acknowledgments, and author contributions, see the supplemental materials.

## Funding support

This work is the result of NIH funding, in whole or in part, and is subject to the NIH Public Access Policy. Through acceptance of this federal funding, the NIH has been given a right to make the work publicly available in PubMed Central.

- NIH grants (DK138818, to PCK, KMSE, AB).
- Global Grants for Gut Health (to PCK).

## Supplementary Material

Supplemental data

Supporting data values

## Figures and Tables

**Figure 1:**
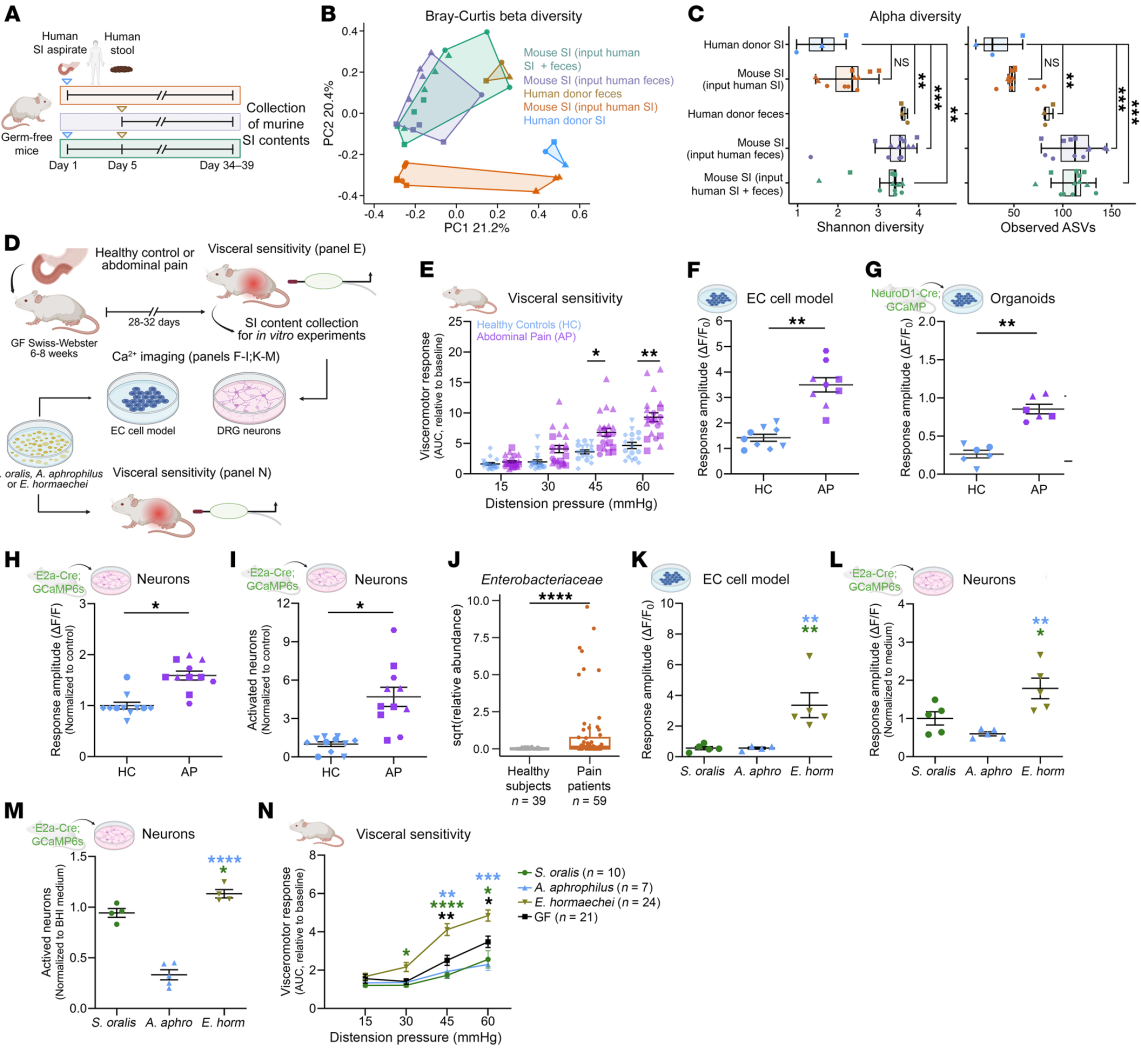
Distinct small intestinal microbes underlie visceral hypersensitivity. (**A**) Experimental design to determine which human sample type best recapitulates the SI microbiome in GF mice (*n* = 3 donor samples, *n* = 3–4 mice/donor/sample site). (**B**) Principal coordinate analysis ordination of Bray-Curtis β-diversity (*n* as in **A**; Supplemental Table 1). (**C**) Shannon diversity and observed ASVs (*n* as in **A**). (**D**) Experimental design to delineate in vitro and in vivo effects of mouse SI aspirates or SI species. (**E**) VMRs to CRD in AP mice (*n* = 4–12 mice/donor, 3 donors) and HC mice (*n* = 6–7 mice/donor, 3 donors). (**F** and **G**) Ca^2+^ response elicited by mouse SI aspirates in QGP-1 cells (Cal 520) and SI organoids (GCaMP) (1–3 SI aspirates/group tested, details in Supplemental Table 3). (**H** and **I**) Amplitude of the Ca^2+^ response and number of neurons activated by mouse SI aspirates in T8–T11 DRG neurons (DRGs from 3–5 mice/aspirate, Supplemental Table 3). (**J**) Relative abundance of *Enterobacteriaceae* in the SI aspirate of patients with symptoms of abdominal pain and bloating. (**K**) Ca^2+^ response evoked by *A*. *aphrophilus*, *S*. *oralis*, and *E*. *hormaechei* supernatants in QGP-1 cells (*n* = 4–5 plates/supernatant, Supplemental Table 5). (**L** and **M**) Ca^2+^ response and number of activated T8–T11 DRG neurons by bacterial supernatants (*n* = 3–5 mice/species, Supplemental Table 5). (**N**) VMRs to CRD in GF mice and GF mice colonized with listed species (*n* = 7–24 mice/group). The following statistical tests were used: ANOVA with Tukey’s HSD (**C**); mixed-effect model corrected for donor (**E**–**I**); Wilcoxon’s rank-sum test with continuity correction (**J**); 1-way ANOVA (**K**–**M**); and repeated measures 2-way ANOVA (**N**). Data are represented as median with 1.5 × IQR (box plots) or mean ± SEM. ****P < 0.0001, ***P < 0.001, **P < 0.01, *P < 0.05.

## Version 1 10/23/2025

In-Press Preview

## Version 2 12/15/2025

Electronic publication
