# Supplementary material for: Distinct human small intestinal microbiome communities underlie visceral hypersensitivity in a humanized mouse model
Source: J Clin Invest. 2025 Oct 23;135(24):e190638. doi: 10.1172/JCI190638 (PMC12700534; doi:10.1172/JCI190638)
Supplement: Supplemental data [file jci-135-190638-s192.pdf]

DISTINCT HUMAN SMALL INTESTINAL MICROBIOME COMMUNITIES UNDERLIE VISCERAL HYPERSENSITIVITY IN A HUMANIZED MOUSE MODEL

SUPPLEMENTARY MATERIAL

Supplementary Table 1. Degree of engraftment following colonization with different human donor samples (*profiling*). Defined analogous to Turnbaugh et al. (1). Genera above 0.1% in human sample also found in any of the mouse samples for that donor. All mouse groups were n=4 except for human SI to mouse SI for sample 3 (square) which had 3 mice.

Input Sample	Number of genera in input sample >0.1%	Number of genera not detected in mouse sample	Number of genera detected in mouse sample	% Engraftment
**Stool to stool engraftment**				
human stool Sample 1 (circle) n=4	41	9	32	78.0
human stool Sample 2 (triangle) n=4	39	7	32	82.1
human stool Sample 3 (square) n=4	48	8	40	83.3
**SI to SI engraftment**				
human SI Sample 1 (circle) n=4	30	9	21	70.0
human SI Sample 2 (triangle) n=4	28	15	13	46.4
human SI Sample 3 (square) n=3	49	18	29	59.2

Supplementary Table 2. Donor demographics and clinical features for abdominal pain experiments (*phenotyping*).

Donor	Symptom status	Age, yr	Sex	BMI, kg/m2	Nosocomial exposure	Pain location	Duration of symptoms	Bloating	Antibiotic use*	Probiotic use*	PPI use
1	Healthy	25	Male	25.9	No	N/A	N/A	No	No	No	No
2	Healthy	66	Male	43.9	No	N/A	N/A	No	No	No	No
3	Healthy	23	Female	23.0	No	N/A	N/A	No	No	Yes	No
4	Symptomatic	54	Female	23.6	Yes	Right-hemiabdomen	3.5-years	Yes	Yes	No	Yes
5	Symptomatic	61	Female	22.4	No	Epigastric	2-years	No	No	No	Yes
6	Symptomatic	69	Female	20.3	No	Epigastric	1-year	Yes	No	No	No

*within the last one month, PPI: proton pump inhibitor.

Supplementary Table 3. Sample sizes for the in vivo and in vitro experiments outlined in Figure 1E-L.

		Visceral sensitivity (panel E)	EC cell model (panel F)	Organoids (panel G)	Neurons (panel H-I)
**Human donor**	**Symbol in figure**	**Number of mice per human donor**	**Number of aspirates (Total number of cells tested per aspirate)**	**Number of aspirates (Total number of cells tested per aspirate)**	**Number of mice (Number of KCl-responding neurons per mouse)**
AP1	▲	12 (6 males, 6 females)	3 (45, 36, 54)	3 (6, 5, 7)	3 (36, 129, 41)
AP2	■	4 (2 males, 2 females)	3 (33, 33, 35)	2 (6,7)	5 (49, 35, 27, 68, 59)
AP3	⬣	6 (3 males, 3 females)	3 (23, 19, 42)	1 (6)	3 (100, 124, 93)
HC1	●	6 (3 males, 3 females)	3 (35, 29, 22)	2 (5, 3)	3 (112, 40, 33)
HC2	▼	4 (2 males, 2 females)	3 (38, 40, 26)	2 (4, 2)	5 (48, 30, 27, 37, 24)
HC3	♦	7 (3 males, 4 females)	3 (32, 28, 30)	2 (3, 3)	3 (60, 102, 95)

Supplementary Table 4. Results from the GTDB-Tk analysis on whole genome of isolated strain (NCBI/Genbank BioSample accession number SAMN50519561).

**User genome**	G2assembly
**Classification**	d__Bacteria;p__Proteobacteria;c__Gammaproteobacteria;o__Enterobacterales;f__Enterobacteriaceae;g__Enterobacter;s__Enterobacter hormaechei_A
**Fastani Reference**	GCF_001729745.1
**Fastani Reference Radius**	95
**Fastani Taxonomy**	d__Bacteria;p__Proteobacteria;c__Gammaproteobacteria;o__Enterobacterales;f__Enterobacteriaceae;g__Enterobacter;s__Enterobacter hormaechei_A
**Fastani Ani**	95.97
**Fastani Af**	0.921
**Closest Placement Reference**	GCF_900322725.1
**Closest Placement Radius**	95
**MSA (%)**	96.78

Supplementary Table 5. Sample sizes for the in vitro EC cell model and neurons experiments outlined in figure 1K-M.

		**EC cell model (panel K)**	**Neurons (panel L-M)**
**Bacterial supernatant**	**Symbol in figure**	**Number of cells tested per plate**	**Number of KCl-responding neurons per mouse**
S. oralis	●	15, 12, 18, 13, 9	40, 35, 40, 36, 35
A. aphrophilus	▲	8, 11, 14, 12	30, 40, 60, 47, 95
E. hormaechei	▼	19, 20, 20, 18, 16	40, 30, 36, 35, 40

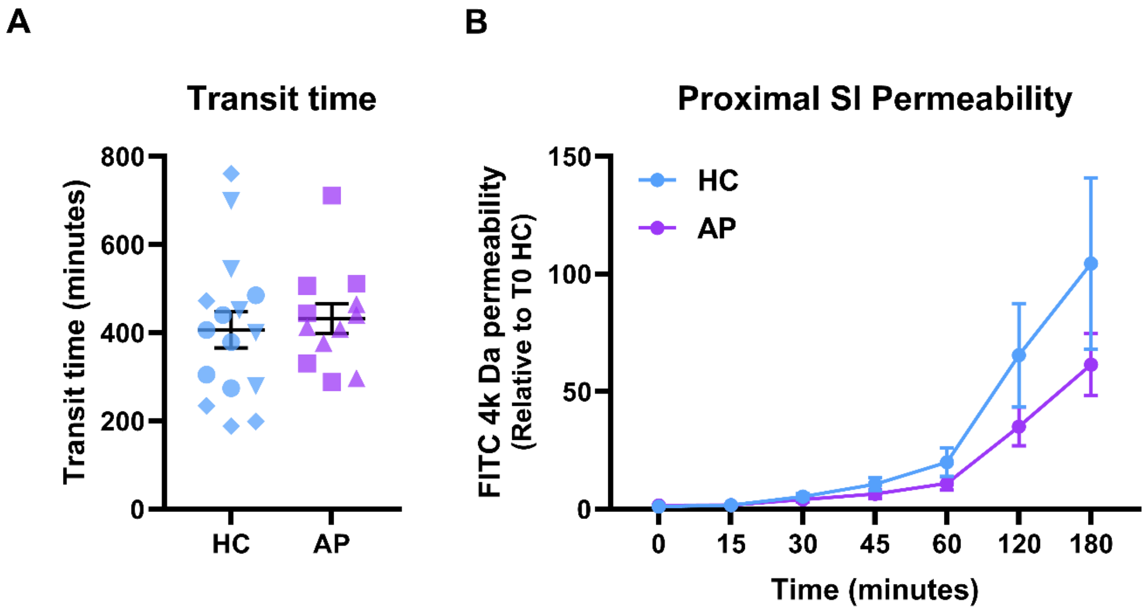

Supplementary Figure 1. Colonization with the SI microbiome from patients with abdominal pain had no effect on whole gut transit time and epithelial permeability in mice. (A) Whole gut transit time (minutes) for abdominal pain (AP) and healthy control (HC) mice (n=4-6 mice/donor). **(B)** Permeability to 4kDa FITC-dextran in proximal small intestine (n=4-12 mice/donor). Data in both panels represent mean ± SEM. Potential differences between groups were assessed with Student's t-test (Panel A) and a repeated measures two-way ANOVA (panel B): ns, $p > 0.05$.

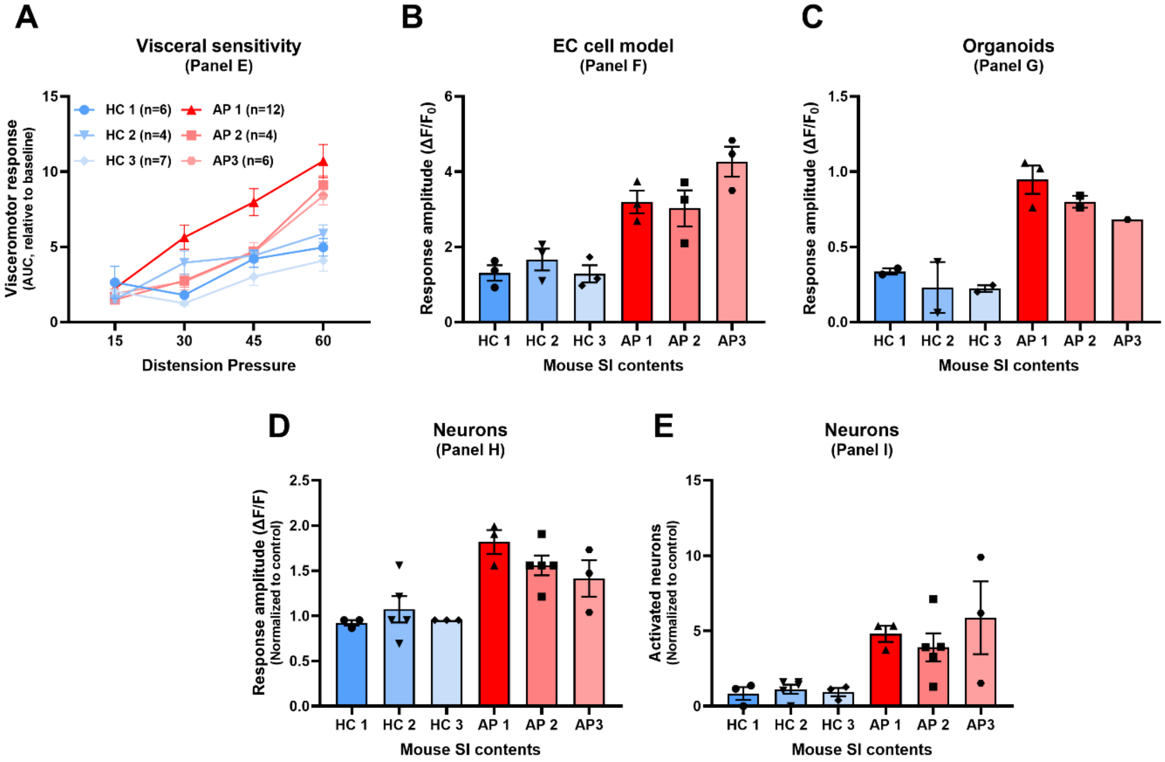

Supplementary Figure 2. HC vs AP group data separated for the individual donors (3 HC and 3 AP). (A) Visceral sensitivity of individual donor groups. Each group consisted of 4-12 mice. Mouse numbers are listed in Supplementary Table 3. (B) Ca^{2+} responses of QGP-1 cells after stimulation with mouse SI aspirates. From each donor group, 3 individual aspirates were tested. Each data point represents the average of 3 plates (7-20 cells/plate). (C) Ca^{2+} responses of organoids after stimulation with mouse SI aspirates. From each donor group, 1-3 aspirates were tested. Each data point represents the average of 2-7 cells. (D-E) Response amplitude and number of responding dissociated T8-T11 DRG neurons after stimulation with mouse SI aspirate. One aspirate from each donor group was selected and tested on DRGs isolated from 3-5 mice. Data in all panels represent mean ± SEM.

METHODS

Sex as a biological variable

All experiments were conducted on male and female mice. We did not observe any sex-related differences. Therefore, male and female mice were grouped during the statistical analysis.

Collection and selection of human samples

Small intestinal aspirates and fecal samples used for profiling experiments were obtained from two healthy donors (26-year-old male and female) enrolled in a diet intervention study (IRB 16-006388). Aspirates from the male donor were collected by EGD both before (sample 1) and after (sample 2) the intervention, while the aspirate from the female donor was collected prior to the intervention (sample 3). Fecal samples from both donors were collected at the time of EGD. For phenotyping experiments, aspirates from three healthy donors (Supplementary Table 2) were obtained from another diet intervention study (IRB 20-008067) in which participants underwent EGD prior to the intervention. Small intestinal aspirates from patients with abdominal pain undergoing EGD were obtained directly from the clinical microbiology laboratory. We selected small intestinal aspirates from healthy controls and patients with abdominal pain in our experiments based on sample availability and only used samples where more than 600 µl was available. Patients were selected based on the availability of sufficient sample for mouse studies, and negative for small intestinal bacterial overgrowth of anaerobes.

Experimental design

Recapitulation of the human SI microbiome in mice (profiling)

Small intestinal aspirates were prepared by mixing an equal volume of SI aspirate with pre-reduced PBS inside an anaerobic chamber (Coy Laboratory Products, Grass Lake, MI). Stool suspensions for gavaging were generated by mixing equal volumes of stool and sterile pre-reduced PBS in a screw cap vial inside the anaerobic chamber. The vials were then sealed before removing from the anaerobic chamber to be transferred to GF isolators. Each unique donor sample was used in three experimental conditions, and for each experimental condition we used 3-4 mice: (1) gavage with SI aspirate only, (2) gavage with stool only and (3) sequential gavage of SI aspirate followed by stool (three days later). Mice were gavaged with 200 µl of the prepared (stool or small intestinal aspirate) suspension and allowed 4-5 weeks for the microbiota to adapt to the mouse gut. At the end of the experimental period, mice were placed in sterilized home cages for pellet collection without access to food or water for one hour. To collect mouse SI contents, mice were first euthanized by CO_2 overdose and two 1 cm sections of the duodenum (2 cm distal from stomach) were dissected out and flushed with 1 ml sterile saline. All SI and pellet samples were flash-frozen in liquid N_2 and stored at -80 C until DNA extraction for sequencing of the microbiome.

Transfer of the GI phenotype from human donors to recipient mice using the SI microbiome (phenotyping)
Three samples from healthy controls and three samples from patients with abdominal pain were selected that met the criteria for sample availability. SI aspirates were prepared as described above and transferred to GF mice. Based on sample availability we were able to gavage 4-12 GF mice per human SI aspirate (the number of mice used per human donor can be found in Supplementary Table 3). Physiological assays (visceral sensitivity and whole gut transit time) were performed 4-5 weeks after gavage with the human SI aspirate. On the day of the physiological assay, mice were placed in sterilized home cages for pellet collection without access to food or water for one hour (whole gut transit time) or four hours (visceral sensitivity). Pellets and SI contents were collected from the mice, as described above, after completion of the physiological assay. SI contents were stored for microbiome profiling and subsequent in vitro experiments on EC cells, SI organoids and dissociated T8-T11 DRG neurons.

Colonization experiments with single bacteria
For mono-colonization experiments *E. hormaechei* from glycerol stock was precultured on HE agar. Single colonies were picked and cultured in LB medium overnight. *S. oralis* and *A. aphrophilus* from glycerol stocks were precultured on chocolate agar and single colonies were grown in BHI medium overnight. GF mice (6-8 weeks) were gavaged with 300 µl of bacterial suspension, containing 1.08 – 1.65 x 10^{10} CFU. Visceral sensitivity was assessed 4-5 weeks after colonization.

Mouse Husbandry
Germ-free Swiss Webster breeding mice (Tac:SW) were purchased from Taconic Farms (Germantown, NY, USA) and experimental mice were bred in the Mayo Clinic gnotobiotic facility. Age-matched male and female germ-free Swiss Webster mice between 6-8 weeks were used for experiments. All mice used in the study were maintained in 12 hr light-dark cycle, were monitored daily for signs of any obvious physical stress and behavioral changes and euthanized per protocol if found in distress. GF mice were maintained in open top cages inside flexible film vinyl isolators (Class Biologically Clean, Madison, WI), while gnotobiotic mice (GF mice colonized with complex community or single bacterium) were maintained in individually ventilated ISOcages (Techniplast, Buguggiate, Italy) in the Mayo Clinic gnotobiotic facility. Mice had ad lib acces to autoclaved diet (Purina Lab diet, 5K67, Collins feed and seed center, Rochester, MN), and autoclaved nanopure water. GF status was confirmed prior to start of experiments with two consecutive cultures of fecal pellet, feed, and bedding on brain heart infusion (BHI), Sabouraud dextrose, and nutrient media under both anaerobic and aerobic condition as well as PCR of 16 s rRNA gene in fecal

DNA using universal primer and *Turicibacter* primer (2). If needed, Gram stain was also performed to further confirm GF status.

E2a-Cre mice (Jax 003724) and RCL-GCaMP6s (Jax 028866) mice were purchased from The Jackson Laboratories (Bar Harbor, Maine, USA) and bred together to generate experimental mice. NeuroD1-Cre mice were a kind gift of Dr. Andrew Leiter (University of Massachusetts, Worcester, MA, USA, since then submitted to The Jackson Laboratories: Jax 028364). Mice were bred with GCaMP5-tdTomato (Jax 024477) to generate experimental mice. E2a-Cre;GCaMP6 mice and NeuroD1-Cre;GCaMP5-tdTomato mice were housed in the conventional animal facility at Mayo Clinic in ventilated cages with a 12-hour light cycle along with ad libitum access to standard chow and water. Housing conditions were approved by the Association for Assessment and Accreditation of Laboratory Animal Care (AAALAC). Male and female mice were used for experiments when 8-10 weeks old.

16S rRNA based profiling of small intestinal microbial communities

Microbial DNA from SI aspirate and fecal samples was extracted using phenol chloroform method. Microbial DNA was sent to the University of Minnesota Genomics Center for 16S rRNA sequencing. Amplicons were generated from the 16S rRNA V4 hypervariable region using barcoded primers ([forward overhang] + 515 F: [TCGTCGGCAGCGTCAGATGTGTATAAGAGACAG]GTGCCAGCMGCCGCGGTAA; and [reverse overhang] + 806R: [GTCTCGTGGGCTCGGAGATGTGTATAAGAGACAG] GGACTACHVGGGTWTCTAAT). Samples were sequenced using Illumina MiSeq using 2x300 bp chemistries. Sequencing reads were annotated essentially as described by Callahan et al. (2016) (3). Briefly, Atropos was used to remove primers, DADA2 for trimming, denoising, taxonomy assignment (with Silva v132 16S sequence database), SortMeRNA and Infernal for filtering, and FastTree for generating phylogenetic trees. All analyses of microbial data, including statistical testing and visualization, were conducted in R (version 4.3.1). Samples with read counts below the median of the negative controls were excluded from further analysis (n=2 samples). Amplicon sequencing variants (ASVs) present in less than 10% of the samples were filtered out. To assess and compare microbial diversity among groups, we rarefied sequence data to the minimum read depth across samples, where appropriate transformed the data to relative abundance, and calculated alpha and beta diversities using the *vegan* package. For differential abundance analysis, a R script calculating U statistics (Mann-Whitney test) was employed to compare mouse samples between pain and the two healthy control groups. Figures were generated with the *ggplot2* and *ape* packages (4, 5).

Bacterial Culturing

Isolation and identification of Enterobacter hormaechei

To identify bacteria uniquely present in the AP mice, we compared the microbial community of AP mice (from one patient with abdominal pain) with HC mice (from two healthy controls). Two amplicon sequencing variants (ASVs) were elevated in AP mice in both comparisons. These were annotated as *Enterobactericeae*_ASV_5 and *Blautia*_ASV27. Of these two, *Enterobactericeae*_ASV_5 was also detected in the original SI aspirate input sample from the patient with abdominal pain. To isolate *Enterobacteriaceae* spp., a murine SI sample with a high relative *Enterobacteriaceae* spp. abundance was plated on Remel Hektoen Enteric agar (Thermo Scientific). Single isolates were obtained through multiple passages. Whole genome sequencing using short read illumina was performed by SeqCoast Genomics (Portsmouth, NH, USA). The Genome Taxonomy Database-Toolkit (GTDB-Tk) was used on the assembled genome to obtain species level identification (8). We additionally screened isolates with MALDI-TOF and confirmed that all isolates belonged to *Enterobacter hormaechei*.

Culturing of *S. oralis* and *A. aphrophilus*

The *Streptococcus oralis* stock was bought from ATCC (#9811, Manassas, Virginia, USA). The *Aggregatibacter aphrophilus* stock was obtained from the Clinical Microbiology laboratory at Mayo Clinic. Both strains were grown on chocolate agar and single colonies were grown statically in BHI medium before gavage or in vitro experiments. *Aggregatibacter aphrophilus* was grown in a CO_2 incubator for 48 hours while *Streptococcus oralis* was grown overnight.

Identification of bacteria using MALDI-TOF

Bacterial isolates were cultured on Hektoen Enteric Agar (ThermoFisher Scientific) and incubated at 37°C for a duration of 24 hours. Following incubation, bacterial cells were harvested from the isolated colonies and spotted onto a MALDI target plate at four distinct locations, allowing the samples to air dry at room temperature. Each spot was subsequently overlaid with 1 µl of Bruker matrix solution (HCCA; α-cyano-4-hydroxycinnamic acid dissolved in a mixture of acetonitrile, water, and trifluoroacetic acid). Additionally, the target plate included a control spot containing pure matrix solution, serving as a negative control, as well as a spot containing the Bacterial Test Standard (Bruker Daltonics GmbH & Co. KG) for calibration purposes. Analysis was conducted using the MALDI Bruker Biotyper Sirius system (Bruker Daltonics GmbH & Co. KG) in an automated fashion. Mass spectra were acquired with FlexControl software (v3.4), in accordance with the manufacturer’s protocols (Bruker Daltonics GmbH & Co. KG, Germany). Species identification was performed by comparing the obtained mass spectra to reference spectra from the BDAL database (Bruker Daltonics GmbH & Co. KG, Germany). Species labels were assigned based on the highest

match identified between the generated mass spectra and those cataloged in the BDAL database. A MALDI-TOF score greater than 2 was established as the threshold for positive species identification.

Visceromotor response graded isobaric colorectal distention

A balloon-pressure sensor was constructed from a PE50 catheter, a balloon (1 cm width x 2 cm length) prepared from a polyethylene plastic, and a miniaturized pressure transducer catheter (SPR-524 Mikro-Tip catheter; Millar Instruments, Houston, TX, USA). The catheter was attached 2 cm below, and the balloon was attached 1 cm below the tip of the pressure sensor. The PE50 catheter was connected to a barostat (Distender Series II, G&J Electronics Inc. Toronto, Canada), and the pressure transducer catheter was connected to a pre-amplifier (PowerLabs 4/35, AD Instruments, Colorado, USA). Voltage-to-pressure output was recorded with LabChart v8 software (AD Instruments, Colorado, USA). Before the start of each experiment, the pressure sensor was calibrated to known pressures of 20 and 60 mmHg. Mice were anesthetized with isoflurane (2% in O2), and the lubricated balloon-pressure sensor was inserted into the colorectum. Once the distal end of the balloon was 0.5 cm past the anal verge, the sensor was securely taped to the tail. Each mouse was placed in an adjustable mouse restrainer (35g, #51325, Stoelting Co, Illinois, USA). The CRD protocol consisted of a set of graded distensions to isobaric pressures of 15, 30, 45, and 60 mmHg. Each pressure was held for 10 seconds, and repeated for three times, with 4-min intervals between pressures.

The tonic smooth muscle contractility signal was removed from the pressure readouts. The visceromotor response (VMR) was defined as the increase in area-under-the-curve (AUC) during each distension. The VMR for each distension was normalized against "baseline" (a 10 sec interval when the distension pressure was 0 mmHg). The average VMR was calculated for each distension pressure and used for data analysis.

Whole-gut transit assay

The automated whole gut transit setup was constructed as described previously (6). Briefly, we used modified cylindrical plastic jars with lids, that had their bottoms replaced with perforated aluminum sheets. These jars were placed on top of Teflon-coated funnels that ended in a 35 mm collection plates filled with 100% glycerol. A high-definition (HD) cameras (TENVIS JPT3815 W-HD) was placed below each collection plate. The setup was surrounded by flexible LED strips, to improve illumination when the cabinet doors were closed. VideoVelocity software was used to record time-lapse videos (1 frame every 10 s).

On the day of the experiment, one hour before the start, food was taken away from the mice. Mice were gavaged with 300 µL carmine solution. Carmine solution was made by dissolving carmine (6% w/v) (Sigma-Aldrich, USA) in methylcellulose (0.5% w/v) (Sigma-Aldrich, USA). After gavage, mice were

placed in the transit chambers. Recordings were started as soon as the mouse was in the transit chambers (T_{start}). Transit time ($T_{transit}$) was calculated by subtracting T_{start} from the time until the appearance of the first red pellet (T_{red}).

Intestinal permeability using Ussing chamber

To measure epithelial FITC-dextran 4kDa permeability, proximal small intestine tissue from mice colonized with human small intestinal microbiota were collected four weeks after initial gavage. The mesentery was removed from a 2-3 cm segment of the small intestine, after which it was opened along the mesenteric border and washed with ice-cold Krebs solution (composition in mM: 10.0 mannitol, 115 NaCl, 2.0 KH2PO4, 2.39 MgCl2*6H2O, 25.0 NaHCO3, 8.0 KCl, 1.3 CaCl2*2H2O; pH 7.3–7.4). The tissue was pinned flat on Sylgard (Dow Corning, Auburn, MI) with the serosal side up so that the muscle layer could be removed. The resulting mucosa-submucosa epithelial sheet was mounted in an Ussing chamber cassette (Physiologic Instruments, San Diego, USA) with an aperture of 0.31 cm^2. The luminal side was bathed with 4 mL of Krebs Mannitol solution (composition in mM: 10.0 Mannitol, 115 NaCl, 2.0 KH2PO4, 2.39 MgCl2*6H2O, 25.0 NaHCO3, 8.0 KCl, 1.3 CaCl2*2H2O; pH 7.3–7.4), while the serosal side of the chamber was bathed with 4 mL of glucose containing Krebs solution (composition in mM: 10.0 D-glucose, 115 NaCl, 2.0 KH2PO4, 2.39 MgCl2*6H2O, 25.0 NaHCO3, 8.0 KCl, 1.3 CaCl2*2H2O; pH 7.3–7.4) while the chamber was bubbled with a 95% O_2 and 5% CO_2 gas mixture. After 30 minutes of equilibration, 100 ul of 10 mg/ml FITC-dextran 4 kDa was added to the luminal side. At 0, 15, 30, 45, 60, 120 and 180 minutes, 3 times 200 ul of solution was removed from the serosal side and replaced with glucose containing Krebs solution. At the end of the experiment, FITC fluorescence was measured at 485 and 528 nm on a BioTek Syngery MX.

Calcium imaging of QGP-1 cells and SI organoids

QGP-1 cells were a kind gift from Dr. Valeria Giandomenico (Uppsala University, Sweden). The QGP-1 cells were cultured in DMEM/F12 supplemented with 10% fetal bovine serum (FBS) and 100 U/mL penicillin/streptomycin at 37°C in a 5% CO2 humidified atmosphere. The QGP-1 cells were cultured in uncoated 35 mm glass dishes (Mattek, Ashland,MA, USA). After reaching 80% confluence, cells were incubated for 20 minutes with 1 µM Cal-520 AM (AAT Bioquest, Sunnyvale, CA, USA) and 0.1% Pluronic F-127 (Invitrogen, Waltham, MA, USA) in serum-free DMEM/F12. The cells were washed with DMEM/F12 serum-free medium. Medium was replaced with HEPES-buffered Ringer (HBR) imaging medium (composition in mM: 5.6 D-glucose, 144 NaCl, 3.0 KCl, 1 MgCl2, 1.5 CaCl2, 10 HEPES; pH 7.4).

SI organoids were derived from NeuroD1Cre-tdTomato/GCaMP mice. After euthanasia, the SI was removed from the abdominal cavity, dissected into small sections and washed 15 times with Ca^{2+} and Mg^{2+}

free Dulbecco's phosphate-buffered saline (DPBS, StemCell Technologies). Intestinal crypts were dissociated with Gentle Cell Dissociation Reagent (GCDR, StemCell Technologies) (15 minutes). After filtration with a 70 µm cell strainer, the suspension was centrifuged at 500g at 4 °C for 5 minutes and resuspended in DMEM. After centrifugation at 500g, at 4 °C for 5 minutes, pelleted crypts were resuspended IntestiCult Organoid Growth Medium (IOGM, StemCell Technologies) and mixed at a 1:1 ratio with Matrigel (Corning). 400 µL domes were plated in pre-warmed 6-well dishes (Corning) and cultured in 3 mL IOGM per well. For calcium imaging experiments, organoids were dissociated with GCDR for 20 minutes, and centrifuged (500g at 4 °C for 5 minutes). After centrifugation, cells were resuspended in DMEM and centrifugated once more. The resulting pellets were seeded on uncoated 35 mm glass dishes (MatTek Life Sciences) for at least 24 hours before imaging. EEC were identified by their TdTomato expression, and these cells were used for subsequent calcium imaging experiments.

Calcium imaging was carried out on an upright Olympus IX70 epifluorescence microscope (Olympus) and imaged with a 16-bit high-speed camera (ORCA-Flash4.0, Hamamatsu), a CoolLED pE-300Ultra illumination system (CoolLED limited, UK), and Metamorph Software (Molecular Devices) for acquisition, all driven by pCLAMP 10 (Molecular Devices). HBR medium was removed from the glass dish before direct application of the SI aspirate to the cells. Cell viability was tested by applying 50 mM KCl to the dish at the end of the experiment. The Ca^{2+} transients were collected at a sampling rate of 300 ms. Cells that responded to the application of the SI aspirate were designated as a Region of Interest (ROI) in MetaMorph (Molecular Devices, San Jose, CA, USA) alongside a background region that did not show any response.

For data analysis, background intensity was subtracted from ROI intensity. Resulting data were normalized against the stable baseline line. The maximum Ca^{2+} response (F_{max}/F_0) to application was determined by subtracting average intensity recorded during the 10 seconds before the application of the SI aspirate (F_0) from the maximal intensity which occurred within 10 seconds after application of the SI aspirate (F_{max}).

Calcium imaging of dissociated DRG neurons

DRGs were collected and dissociated as described previously (7). For the preparation of dissociated neurons, E2-Cre;GCamp6 mice were transcardially perfused with cold Ca^{2+}/Mg^{2+}-free HBSS (Thermofisher #14170161), DRG (T9-T12) were removed and placed in DMEM F-12 (10% fetal bovine serum and 1% penicillin/streptomycin), and enzymatically treated with cysteine (Sigma, #C7352-25G), papain (Worthington Biochemical corporation, #LS003126), collagenase type II (Worthington Biochemical corporation, #LS003126), and dispase type II (Sigma-Aldrich, #D4693-1G). Ganglia were dissociated with

mechanical trituration and neurons were plated on 35mmx10mm laminin/poly-d-lysine coverslips for 2 hours and then flooded with additional media. For the preparation of intact neurons, E2-Cre;GCamp6 mice were transcardially perfused with cold Ca^{2+}/Mg^{2+}-free HBSS (Thermofisher #14170161), DRG (T9-T12) were removed and placed in DMEM F-12 (10% fetal bovine serum and 1% penicillin/streptomycin).

For each coverslip, GCaMP recordings of DRG neuron activity were captured for one minute during baseline and followed by another one-minute recording after application of SI aspirate from a mouse colonized with healthy control SI material or SI aspirate from mice colonized with SI material from patients with abdominal pain. GCaMP signals were collected using Metamorph Software (Molecular Devices, San Jose, CA) at 20 frames/sec on an upright DM6FS Leica fluorescent microscope (Leica, Deerfield, IL) equipped with a Prime 95B Scientific Complementary Metal-Oxide-Semiconductor (sCMOS) camera (Photometrics, Tucson, AZ) and 20X objective lens. Circular ROIs were placed around all DRG neurons that displayed responses to 50mM KCl as a positive control, and the peak amplitude of GCaMP signals (dF/F0) were calculated for each condition. Neurons were characterized as responders if the change in activity was >200% compared to baseline.

Experimental rigor and statistical analysis.

All analyses of data, including statistical testing and visualization, were conducted in R (version 4.3.1) or GraphPad Prism v10.3.0.

Mouse group sizes in the phenotyping experiments were determined by the availability of the human SI aspirate. Experimenters were blinded to the SI aspirates that were given to the GF mice. Visceral sensitivity was analyzed with a mixed effect model using the human donor as a random effect. We applied a mixed effect model, in which the mouse SI content was a random effect, to analyze the data from calcium imaging experiments (in organoids, QGP-1 cells and dissociated DRG neurons). Visceral hypersensitivity after monocolonization was analyzed using a repeated measures two-way ANOVA with Bonferroni post-hoc test. Calcium imaging data from the experiments involving bacterial supernatants (QGP-1 cells and dissociated DRG neurons) were analyzed with a One-Way ANOVA. $p<0.05$ was considered statistically significant.

STUDY APPROVAL

All human samples were obtained as part of previously published or ongoing studies approved by the Mayo Clinic Institutional Review Board IRB in compliance with all relevant ethical regulations. The human small intestinal samples used for the profiling and phenotyping experiment were collected under IRB protocols IRB16-006388, IRB 20-008067 and IRB 15-003235.

All animal studies were approved by Mayo Clinic Institutional Animal Care and Use Committee (Protocol no. A0006269-21 and A00006603-22-R25) and conducted in compliance with regulatory guidelines.

DATA AVAILABILITY

The raw data for the main figure and supplementary figure is accessible in the accompanying **Supporting Data Values** file. Raw sequencing data have been deposited in the NCBI Sequence Read Archive under BioProject accession number PRJNA1308628. Processed data and R code for microbiome analysis is available at https://github.com/kashyap-microbiome/SI_transplant. Previously published software packages and versions used to analyze our data are cited in the methods above. The assembled *Enterobacter hormaechei* whole genome has been deposited at DDBJ/ENA/GenBank under the accession JBQJIW000000000 (https://www.ncbi.nlm.nih.gov/nuccore/JBQJIW000000000).

ACKNOWLEDGEMENTS

This work is the result of NIH funding, in whole or in part, and is subject to the NIH Public Access Policy. Through acceptance of this federal funding, the NIH has been given a right to make the work publicly available in PubMed Central. This work was supported by funding from NIH (DK138818, PCK, KSE, AB) and Global Grants for Gut Health (PCK).
Figure 1 panels A and D were created with BioRender.com.

AUTHOR CONTRIBUTIONS

P.C.K. conceptualized the study. I.Y.C., L.T., D.T., Y.X. performed the mouse colonization experiments. T.L., S.B., A.M.P., D.R.L., P.K.S., A.W., B.E. and K.S.E. performed Ca^{2+} imaging experiments. I.Y.C, R.A.T.M and R.S.C. analyzed the microbiome data, T.L, R.A.T.M, Y.X., K.S.E, G.F., A.B., and K.S.E, analyzed the in vitro and in vivo physiology experiments. T.L., I.Y.C., R.A.T.M and P.C.K. drafted the manuscript with input from A.B., K.S.E., G.F., and D.R.L and all authors reviewed and approved the manuscript.

REFERENCES

1. Turnbaugh PJ, Ridaura VK, Faith JJ, Rey FE, Knight R, and Gordon JI. The effect of diet on the human gut microbiome: a metagenomic analysis in humanized gnotobiotic mice. *Sci Transl Med.* 2009;1(6):6ra14.
2. Bhattarai Y, and Kashyap PC. Germ-Free Mice Model for Studying Host-Microbial Interactions. *Methods Mol Biol.* 2016;1438:123-35.

3. Callahan BJ, Sankaran K, Fukuyama JA, McMurdie PJ, and Holmes SP. Bioconductor Workflow for Microbiome Data Analysis: from raw reads to community analyses. *F1000Res.* 2016;5:1492.
4. Paradis E, and Schliep K. ape 5.0: an environment for modern phylogenetics and evolutionary analyses in R. *Bioinformatics.* 2019;35(3):526-8.
5. Wickham H. *Use R!,*. Cham: Springer International Publishing : Imprint: Springer,; 2016:1 online resource (XVI, 260 pages 32 illustrations, 140 illustrations in color.
6. Kacmaz H, Alto A, Knutson K, Linden DR, Gibbons SJ, Farrugia G, et al. A simple automated approach to measure mouse whole gut transit. *Neurogastroenterol Motil.* 2021;33(2):e13994.
7. Malin SA, Davis BM, and Molliver DC. Production of dissociated sensory neuron cultures and considerations for their use in studying neuronal function and plasticity. *Nat Protoc.* 2007;2(1):152-60.
